# Crystal structure of 2-[2-(benz­yloxy)benzyl­idene]malono­nitrile

**DOI:** 10.1107/S2056989015012608

**Published:** 2015-07-08

**Authors:** Sammer Yousuf, Huma Bano, Munira Taj Muhammad, Khalid Mohammed Khan

**Affiliations:** a H.E.J. Research Institute of Chemistry, International Center for Chemical and Biological Sciences, University of Karachi, Karachi 75270, Pakistan

**Keywords:** crystal structure, malono­nitrile, benzyl­idenemalono­nitrile derivatives

## Abstract

In the title benzyl­idenemalono­nitrile derivative, C_17_H_12_N_2_O, the dihedral angles between the central benzene ring and the Y-shaped C=C(CN)_2_ group (r.m.s. deviation = 0.006 Å) and the terminal benzene ring are 12.72 (8) and 37.60 (11)°, respectively. The C_ar_—O—C*sp* ^3^—C_ar_ torsion angle is −174.52 (13)° and the major twist between the aromatic rings occurs about the C*sp* ^3^—C_ar_ bond. Weak aromatic π–π stacking [centroid–centroid separation = 3.7784 (13) Å; slippage = 1.21 Å] between inversion-related pairs of the central benzene rings is observed in the crystal.

## Related literature   

For the applications and biological activities of benzyl­idenemalono­nitrile derivatives, see: Turpaev *et al.* (2011 ▸); Sagara *et al.* (2002 ▸); Novogrodsky *et al.* (1994 ▸); Gazit *et al.* (1989 ▸). For the crystal structure of a related compound, see: Gan *et al.* (2012 ▸).
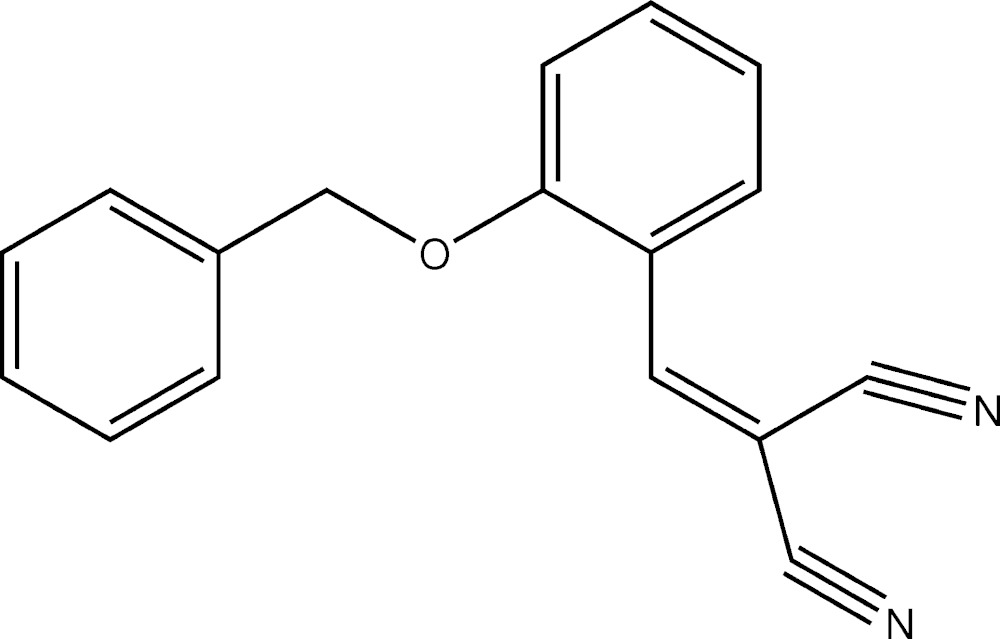


## Experimental   

### Crystal data  

C_17_H_12_N_2_O
*M* *_r_* = 260.29
Triclinic, 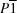

*a* = 7.2959 (9) Å
*b* = 9.4963 (12) Å
*c* = 11.0280 (14) Å
α = 97.709 (3)°
β = 107.953 (3)°
γ = 105.155 (3)°
*V* = 682.13 (15) Å^3^
*Z* = 2
Mo *K*α radiation
μ = 0.08 mm^−1^
*T* = 293 K
0.34 × 0.11 × 0.07 mm

### Data collection  

Bruker SMART APEX CCD diffractometer
Absorption correction: multi-scan (*SADABS*; Bruker, 2000 ▸) *T*_min_ = 0.973, *T*_max_ = 0.994
7776 measured reflections
2545 independent reflections
1722 reflections with *I* > 2σ(*I*)
*R*_int_ = 0.032

### Refinement  

*R*[*F* ^2^ > 2σ(*F* ^2^)] = 0.045
*wR*(*F* ^2^) = 0.112
*S* = 1.01
2545 reflections
181 parameters
H-atom parameters constrained
Δρ_max_ = 0.11 e Å^−3^
Δρ_min_ = −0.17 e Å^−3^

### 

Data collection: *SMART* (Bruker, 2000 ▸); cell refinement: *SAINT* (Bruker, 2000 ▸); data reduction: *SAINT*; program(s) used to solve structure: *SHELXS97* (Sheldrick, 2008 ▸); program(s) used to refine structure: *SHELXL97* (Sheldrick, 2008 ▸); molecular graphics: *SHELXTL* (Sheldrick, 2008 ▸); software used to prepare material for publication: *SHELXTL*.

## Supplementary Material

Crystal structure: contains datablock(s) global, I. DOI: 10.1107/S2056989015012608/hb7442sup1.cif

Structure factors: contains datablock(s) I. DOI: 10.1107/S2056989015012608/hb7442Isup2.hkl

Supporting information file. DOI: 10.1107/S2056989015012608/hb7442Isup3.cml

**Fig. 1.**
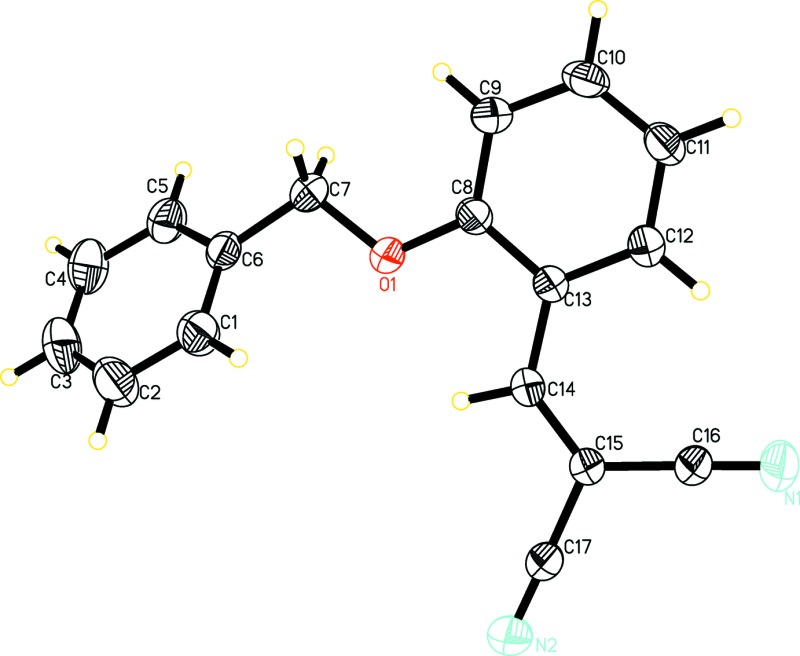
The molecular structure of (I) with displacement ellipsoids drawn at 30% probability level.

. DOI: 10.1107/S2056989015012608/hb7442fig1.tif
The mol­ecular structure of (I) with displacement ellipsoids drawn at 30% probability level.

**Fig. 2.**
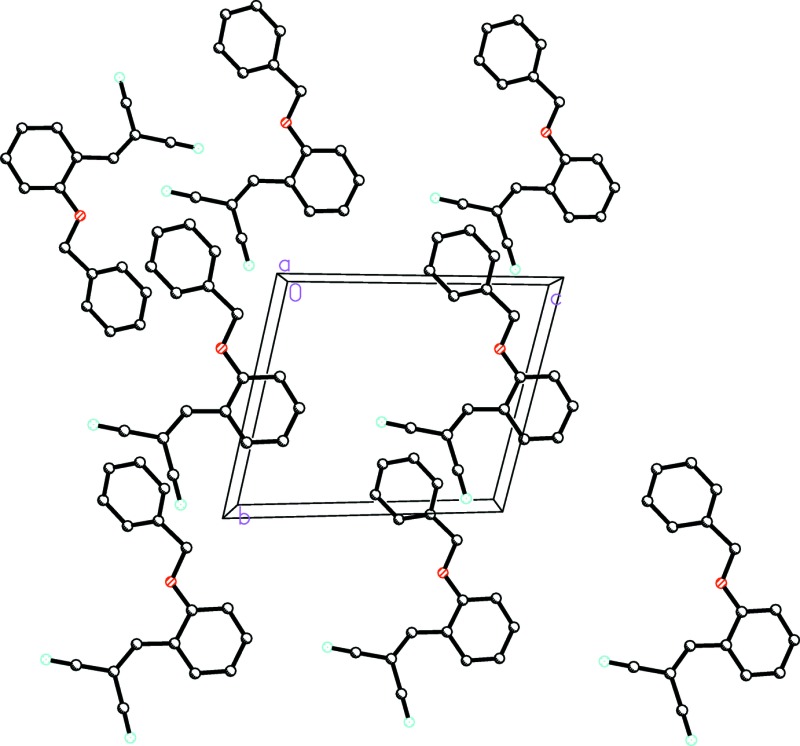
The crystal packing of the title compound (I).

. DOI: 10.1107/S2056989015012608/hb7442fig2.tif
The crystal packing of the title compound (I).

CCDC reference: 1409734

Additional supporting information:  crystallographic information; 3D view; checkCIF report

## supplementary crystallographic information

### S1. Comment

Malononitriles and their benzylidene derivative represent a wide group of organic compounds having a number of pharmacological activities including inhibition of epidermal growth factor protein tyrosine kinas (Turpaev *et al.*, 2011, Gazit *et al.*, 1989), expression of iNOS and COX-2 pro-inflammatory agents. Structural analogues of benzylidenemalononitrile are also known to have free radical scavenging (Sagara *et al.*, 2002) and antiinflammatory properties (suppression of TNFα release) (Novogrodsky *et al.*, 1994). The title compound was obtained as a part of our ongoing resaerch to synthesize and evaluate the biological activities of structural analogues having benzylidenemalononitrile as basic nucleus.

The structure of title compound is similar to that of previously published 2-[4-(benzyloxy)benzylidene]malononitrile (Gan *et al.*, 2012) with the difference that the benzyloxy (O1/C1–C7) group found to be attached at *ortho* position on benzylidenemalononitrile (N1/N2/C8–C16) moiety (Fig. 1) in contrast to *para* position, as observed in previously published 2-[4-(Benzyloxy)benzylidene]malononitrile. The dihedral angles between two planner phenyl rings phenyl(C1–C6)and (C8–C13) is 37.60 (11)°. Dicyanoethylene (N1–N2/C14–C17) group found to be coplanar with the benzene ring (C8–C13) to which it is attached. The bond lengths and angle were found to be similar as in structurally related 2-[4-(benzyloxy)benzylidene]malononitrile (Gan *et al.*, 2012).

### S2. Experimental

In a round-bottomed flask 2-benzyloxybenzaldehyde (1 mmol) and a catalytic amount (3 mol%) of Bi(NO3)_3_ in water/ethanol (10 ml) were stirred for 2 minutes at room temperature followed by the additon of malononitrile (1.1 mmol). The reaction mixture was refluxed for 20 minutes. After completion of the reaction (TLC analysis), Bi(NO_3_)_3_ was filtered for the next use and the filtrate was kept at room temperature over night to obtain crystals. Crystals were filtered, washed with water, dried, and re-crystallized from hot ethanol as colourless plates. Thin layer chromatography was carried out on aluminium plates pre-coated with silica gel (Kieselgel 60, E. Merck, Darmstadt, Germany). UV light at 254 and 365 nm was used for chromatograms visualization.

### S3. Refinement

H atoms on phenyl and methine were positioned geometrically with C—H = 0.93 Å (CH phenyl) and 0.97 Å (CH) and constrained to ride on their parent atoms with *U*_iso_(H)=1.2*U*_eq_(CH).

### Crystal data

**Table:**

C_17_H_12_N_2_O	*Z* = 2
*M**_r_* = 260.29	*F*(000) = 272
Triclinic, *P*1	*D*_x_ = 1.267 Mg m^−^^3^
*a* = 7.2959 (9) Å	Mo *K*α radiation, λ = 0.71073 Å
*b* = 9.4963 (12) Å	Cell parameters from 1299 reflections
*c* = 11.0280 (14) Å	θ = 2.3–20.8°
α = 97.709 (3)°	µ = 0.08 mm^−^^1^
β = 107.953 (3)°	*T* = 293 K
γ = 105.155 (3)°	Plate, colourless
*V* = 682.13 (15) Å^3^	0.34 × 0.11 × 0.07 mm

### Data collection

**Table:**

Bruker SMART APEX CCD diffractometer	2545 independent reflections
Radiation source: fine-focus sealed tube	1722 reflections with *I* > 2σ(*I*)
Graphite monochromator	*R*_int_ = 0.032
ω scan	θ_max_ = 25.5°, θ_min_ = 2.0°
Absorption correction: multi-scan (*SADABS*; Bruker, 2000)	*h* = −8→8
*T*_min_ = 0.973, *T*_max_ = 0.994	*k* = −11→11
7776 measured reflections	*l* = −13→13

### Refinement

**Table:**

Refinement on *F*^2^	Primary atom site location: structure-invariant direct methods
Least-squares matrix: full	Secondary atom site location: difference Fourier map
*R*[*F*^2^ > 2σ(*F*^2^)] = 0.045	Hydrogen site location: inferred from neighbouring sites
*wR*(*F*^2^) = 0.112	H-atom parameters constrained
*S* = 1.01	*w* = 1/[σ^2^(*F*_o_^2^) + (0.0495*P*)^2^ + 0.016*P*] where *P* = (*F*_o_^2^ + 2*F*_c_^2^)/3
2545 reflections	(Δ/σ)_max_ < 0.001
181 parameters	Δρ_max_ = 0.11 e Å^−^^3^
0 restraints	Δρ_min_ = −0.17 e Å^−^^3^

### Special details

**Table:**

Geometry. All e.s.d.'s (except the e.s.d. in the dihedral angle between two l.s. planes) are estimated using the full covariance matrix. The cell e.s.d.'s are taken into account individually in the estimation of e.s.d.'s in distances, angles and torsion angles; correlations between e.s.d.'s in cell parameters are only used when they are defined by crystal symmetry. An approximate (isotropic) treatment of cell e.s.d.'s is used for estimating e.s.d.'s involving l.s. planes.
Refinement. Refinement of *F*^2^ against ALL reflections. The weighted *R*-factor *wR* and goodness of fit *S* are based on *F*^2^, conventional *R*-factors *R* are based on *F*, with *F* set to zero for negative *F*^2^. The threshold expression of *F*^2^ > σ(*F*^2^) is used only for calculating *R*-factors(gt) *etc*. and is not relevant to the choice of reflections for refinement. *R*-factors based on *F*^2^ are statistically about twice as large as those based on *F*, and *R*- factors based on ALL data will be even larger.

### Fractional atomic coordinates and isotropic or equivalent isotropic displacement parameters (Å^2^)

**Table:**

	*x*	*y*	*z*	*U*_iso_*/*U*_eq_	
O1	0.37248 (18)	0.70351 (12)	0.15139 (10)	0.0558 (3)	
N1	−0.0533 (3)	0.06530 (19)	0.15041 (17)	0.0861 (6)	
N2	0.2682 (3)	0.38995 (18)	0.52272 (16)	0.0799 (6)	
C1	0.6578 (3)	0.9358 (2)	0.36642 (19)	0.0682 (6)	
H1A	0.6704	0.8409	0.3666	0.082*	
C2	0.7541 (3)	1.0476 (3)	0.4799 (2)	0.0840 (7)	
H2A	0.8300	1.0276	0.5565	0.101*	
C3	0.7382 (4)	1.1883 (3)	0.4800 (2)	0.0864 (7)	
H3A	0.8036	1.2639	0.5565	0.104*	
C4	0.6260 (3)	1.2171 (2)	0.3675 (2)	0.0816 (7)	
H4A	0.6163	1.3127	0.3670	0.098*	
C5	0.5273 (3)	1.1043 (2)	0.2549 (2)	0.0653 (5)	
H5A	0.4487	1.1241	0.1792	0.078*	
C6	0.5432 (3)	0.96316 (18)	0.25284 (17)	0.0501 (4)	
C7	0.4489 (3)	0.84695 (18)	0.12655 (17)	0.0573 (5)	
H7A	0.3388	0.8708	0.0666	0.069*	
H7B	0.5495	0.8453	0.0863	0.069*	
C8	0.2956 (2)	0.57991 (18)	0.05089 (15)	0.0459 (4)	
C9	0.2793 (3)	0.5848 (2)	−0.07670 (16)	0.0545 (5)	
H9A	0.3218	0.6764	−0.0978	0.065*	
C10	0.2002 (3)	0.4537 (2)	−0.17214 (17)	0.0607 (5)	
H10A	0.1902	0.4575	−0.2577	0.073*	
C11	0.1356 (3)	0.3172 (2)	−0.14330 (17)	0.0609 (5)	
H11A	0.0824	0.2293	−0.2088	0.073*	
C12	0.1501 (3)	0.31157 (19)	−0.01748 (16)	0.0539 (5)	
H12A	0.1061	0.2189	0.0016	0.065*	
C13	0.2296 (2)	0.44161 (17)	0.08304 (15)	0.0440 (4)	
C14	0.2524 (2)	0.44280 (18)	0.21768 (15)	0.0478 (4)	
H14A	0.3315	0.5343	0.2765	0.057*	
C15	0.1784 (2)	0.33340 (18)	0.27265 (15)	0.0473 (4)	
C16	0.0496 (3)	0.1845 (2)	0.20382 (17)	0.0568 (5)	
C17	0.2273 (3)	0.36414 (19)	0.41193 (19)	0.0569 (5)	

### Atomic displacement parameters (Å^2^)

**Table:**

	*U*^11^	*U*^22^	*U*^33^	*U*^12^	*U*^13^	*U*^23^
O1	0.0745 (8)	0.0390 (7)	0.0461 (7)	0.0046 (6)	0.0232 (6)	0.0091 (5)
N1	0.1044 (15)	0.0552 (11)	0.0784 (12)	−0.0080 (10)	0.0388 (11)	0.0033 (9)
N2	0.1137 (15)	0.0664 (11)	0.0508 (11)	0.0125 (10)	0.0309 (10)	0.0144 (9)
C1	0.0809 (14)	0.0509 (12)	0.0639 (13)	0.0202 (10)	0.0152 (11)	0.0126 (10)
C2	0.0897 (17)	0.0751 (16)	0.0632 (14)	0.0207 (13)	0.0055 (12)	0.0023 (12)
C3	0.0827 (16)	0.0622 (15)	0.0862 (17)	0.0083 (12)	0.0189 (13)	−0.0159 (12)
C4	0.0847 (16)	0.0464 (12)	0.1071 (19)	0.0192 (11)	0.0332 (14)	0.0030 (13)
C5	0.0673 (13)	0.0491 (12)	0.0790 (14)	0.0183 (10)	0.0248 (11)	0.0177 (10)
C6	0.0521 (10)	0.0404 (10)	0.0562 (11)	0.0086 (8)	0.0221 (9)	0.0121 (8)
C7	0.0675 (12)	0.0448 (10)	0.0564 (11)	0.0114 (9)	0.0205 (9)	0.0190 (9)
C8	0.0448 (10)	0.0475 (10)	0.0404 (9)	0.0099 (8)	0.0146 (8)	0.0060 (8)
C9	0.0587 (11)	0.0578 (11)	0.0459 (10)	0.0130 (9)	0.0207 (9)	0.0156 (9)
C10	0.0634 (12)	0.0778 (14)	0.0382 (10)	0.0178 (10)	0.0198 (9)	0.0113 (10)
C11	0.0663 (12)	0.0603 (12)	0.0443 (11)	0.0099 (10)	0.0189 (9)	−0.0025 (9)
C12	0.0577 (11)	0.0464 (10)	0.0484 (11)	0.0070 (9)	0.0179 (9)	0.0039 (8)
C13	0.0444 (9)	0.0430 (9)	0.0389 (9)	0.0090 (7)	0.0128 (7)	0.0066 (7)
C14	0.0526 (10)	0.0394 (9)	0.0424 (10)	0.0084 (8)	0.0124 (8)	0.0044 (7)
C15	0.0540 (10)	0.0416 (10)	0.0410 (9)	0.0092 (8)	0.0163 (8)	0.0068 (8)
C16	0.0674 (12)	0.0461 (11)	0.0522 (11)	0.0064 (10)	0.0250 (9)	0.0108 (9)
C17	0.0716 (13)	0.0442 (11)	0.0508 (12)	0.0083 (9)	0.0242 (10)	0.0123 (9)

### Geometric parameters (Å, º)

**Table:**

O1—C8	1.3580 (18)	C7—H7B	0.9700
O1—C7	1.4277 (18)	C8—C9	1.383 (2)
N1—C16	1.138 (2)	C8—C13	1.408 (2)
N2—C17	1.140 (2)	C9—C10	1.374 (2)
C1—C6	1.375 (2)	C9—H9A	0.9300
C1—C2	1.378 (3)	C10—C11	1.374 (2)
C1—H1A	0.9300	C10—H10A	0.9300
C2—C3	1.371 (3)	C11—C12	1.368 (2)
C2—H2A	0.9300	C11—H11A	0.9300
C3—C4	1.366 (3)	C12—C13	1.397 (2)
C3—H3A	0.9300	C12—H12A	0.9300
C4—C5	1.377 (3)	C13—C14	1.440 (2)
C4—H4A	0.9300	C14—C15	1.348 (2)
C5—C6	1.373 (2)	C14—H14A	0.9300
C5—H5A	0.9300	C15—C16	1.427 (2)
C6—C7	1.494 (2)	C15—C17	1.435 (2)
C7—H7A	0.9700		
			
C8—O1—C7	118.82 (13)	O1—C8—C13	115.91 (14)
C6—C1—C2	120.69 (18)	C9—C8—C13	120.32 (15)
C6—C1—H1A	119.7	C10—C9—C8	119.76 (17)
C2—C1—H1A	119.7	C10—C9—H9A	120.1
C3—C2—C1	120.0 (2)	C8—C9—H9A	120.1
C3—C2—H2A	120.0	C9—C10—C11	121.12 (16)
C1—C2—H2A	120.0	C9—C10—H10A	119.4
C4—C3—C2	119.8 (2)	C11—C10—H10A	119.4
C4—C3—H3A	120.1	C12—C11—C10	119.45 (16)
C2—C3—H3A	120.1	C12—C11—H11A	120.3
C3—C4—C5	119.9 (2)	C10—C11—H11A	120.3
C3—C4—H4A	120.0	C11—C12—C13	121.61 (16)
C5—C4—H4A	120.0	C11—C12—H12A	119.2
C6—C5—C4	121.0 (2)	C13—C12—H12A	119.2
C6—C5—H5A	119.5	C12—C13—C8	117.74 (15)
C4—C5—H5A	119.5	C12—C13—C14	124.20 (15)
C5—C6—C1	118.55 (17)	C8—C13—C14	118.05 (14)
C5—C6—C7	119.67 (17)	C15—C14—C13	130.76 (15)
C1—C6—C7	121.64 (16)	C15—C14—H14A	114.6
O1—C7—C6	109.28 (14)	C13—C14—H14A	114.6
O1—C7—H7A	109.8	C14—C15—C16	125.63 (15)
C6—C7—H7A	109.8	C14—C15—C17	119.51 (15)
O1—C7—H7B	109.8	C16—C15—C17	114.86 (14)
C6—C7—H7B	109.8	N1—C16—C15	179.11 (19)
H7A—C7—H7B	108.3	N2—C17—C15	179.2 (2)
O1—C8—C9	123.76 (15)		
			
C6—C1—C2—C3	−0.7 (3)	C9—C10—C11—C12	0.1 (3)
C1—C2—C3—C4	0.2 (4)	C10—C11—C12—C13	−0.1 (3)
C2—C3—C4—C5	0.8 (4)	C11—C12—C13—C8	−0.3 (2)
C3—C4—C5—C6	−1.4 (3)	C11—C12—C13—C14	−178.75 (16)
C4—C5—C6—C1	0.9 (3)	O1—C8—C13—C12	−179.77 (14)
C4—C5—C6—C7	−174.84 (18)	C9—C8—C13—C12	0.6 (2)
C2—C1—C6—C5	0.2 (3)	O1—C8—C13—C14	−1.2 (2)
C2—C1—C6—C7	175.84 (19)	C9—C8—C13—C14	179.23 (15)
C8—O1—C7—C6	−174.52 (13)	C12—C13—C14—C15	−12.2 (3)
C5—C6—C7—O1	−144.67 (16)	C8—C13—C14—C15	169.29 (17)
C1—C6—C7—O1	39.7 (2)	C13—C14—C15—C16	−1.5 (3)
C7—O1—C8—C9	−2.6 (2)	C13—C14—C15—C17	179.08 (17)
C7—O1—C8—C13	177.82 (14)	C14—C15—C16—N1	−176 (100)
O1—C8—C9—C10	179.76 (15)	C17—C15—C16—N1	3 (14)
C13—C8—C9—C10	−0.7 (2)	C14—C15—C17—N2	−22 (16)
C8—C9—C10—C11	0.3 (3)	C16—C15—C17—N2	159 (16)

## References

[bb1] Bruker (2000). *SADABS*, *SMART* and *SAINT*. Bruker AXS Inc., Madison, Wisconsin, USA.

[bb2] Gan, H., Liu, X., Fang, Z. & Guo, K. (2012). *Acta Cryst.* E**68**, o1690. 10.1107/S1600536812020053 PMC3379286 22719484

[bb3] Gazit, A., Yaish, P., Gilon, C. & Levitzki, A. (1989). *J. Med. Chem.* **32**, 2344–2352. 10.1021/jm00130a020 2552117

[bb4] Novogrodsky, A., Vanichkin, A., Patya, M., Gazit, A., Osherov, N. & Levitzki, A. (1994). *Science*, **264**, 1319–1322. 10.1126/science.8191285 8191285

[bb5] Sagara, Y., Ishige, K., Tsai, C. & Maher, P. (2002). *J. Biol. Chem.* **277**, 36204–36215. 10.1074/jbc.M203895200 12121989

[bb6] Sheldrick, G. M. (2008). *Acta Cryst.* A**64**, 112–122. 10.1107/S0108767307043930 18156677

[bb7] Turpaev, K., Ermolenko, M., Cresteil, T. & Drapier, J. C. (2011). *Biochem. Pharmacol.* **82**, 535–547. 10.1016/j.bcp.2011.05.028 21669191

