# Supplementary figures and images for: Crystal structure of 2-[2-(benz­yloxy)benzyl­idene]malono­nitrile
Source: Acta Crystallogr E Crystallogr Commun. 2015 Jul 8;71(Pt 8):o560–1. doi: 10.1107/S2056989015012608 (PMC4571396; doi:10.1107/S2056989015012608)

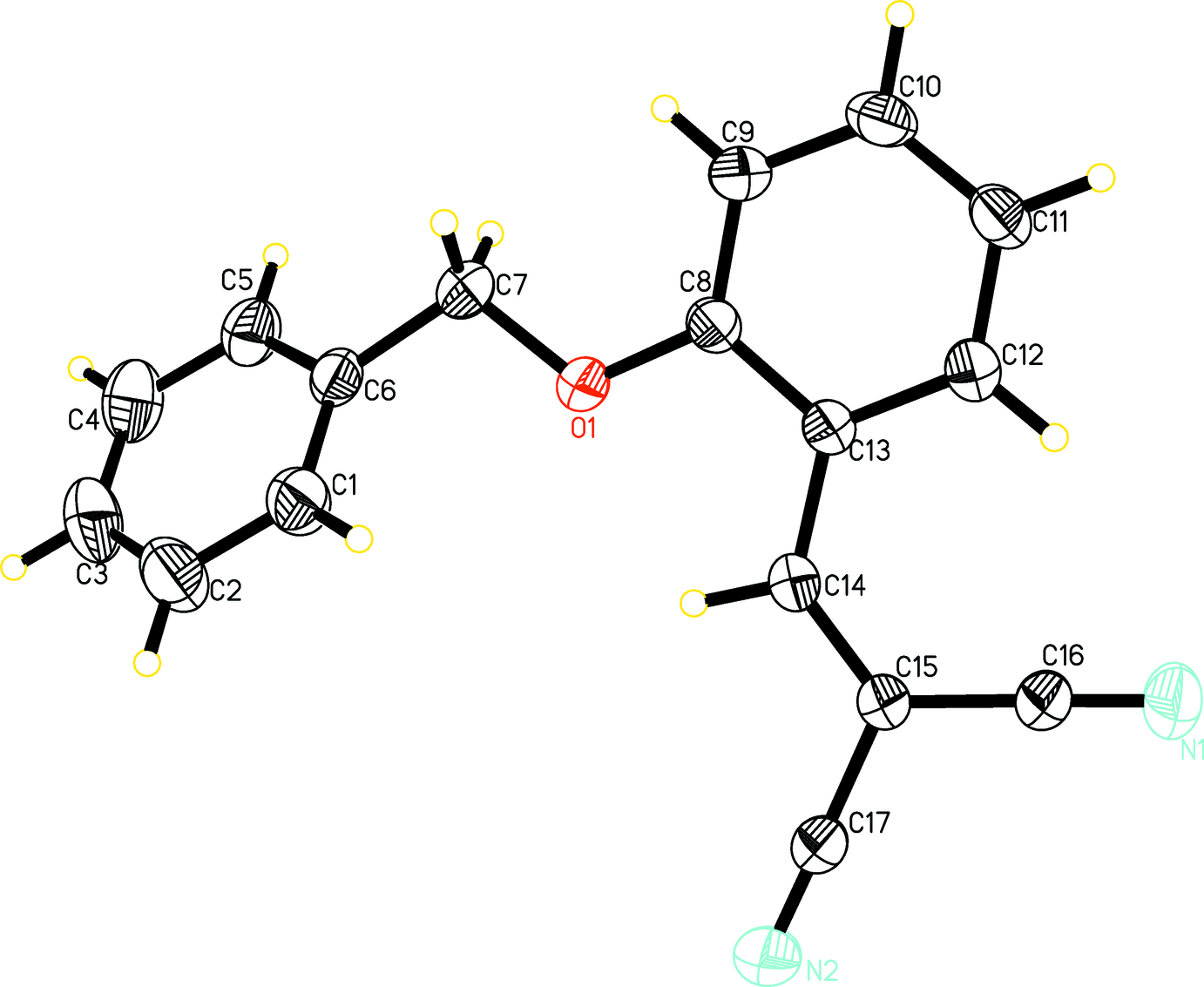

Supplement: Supplementary file 4 [file e-71-0o560-fig1.tif]

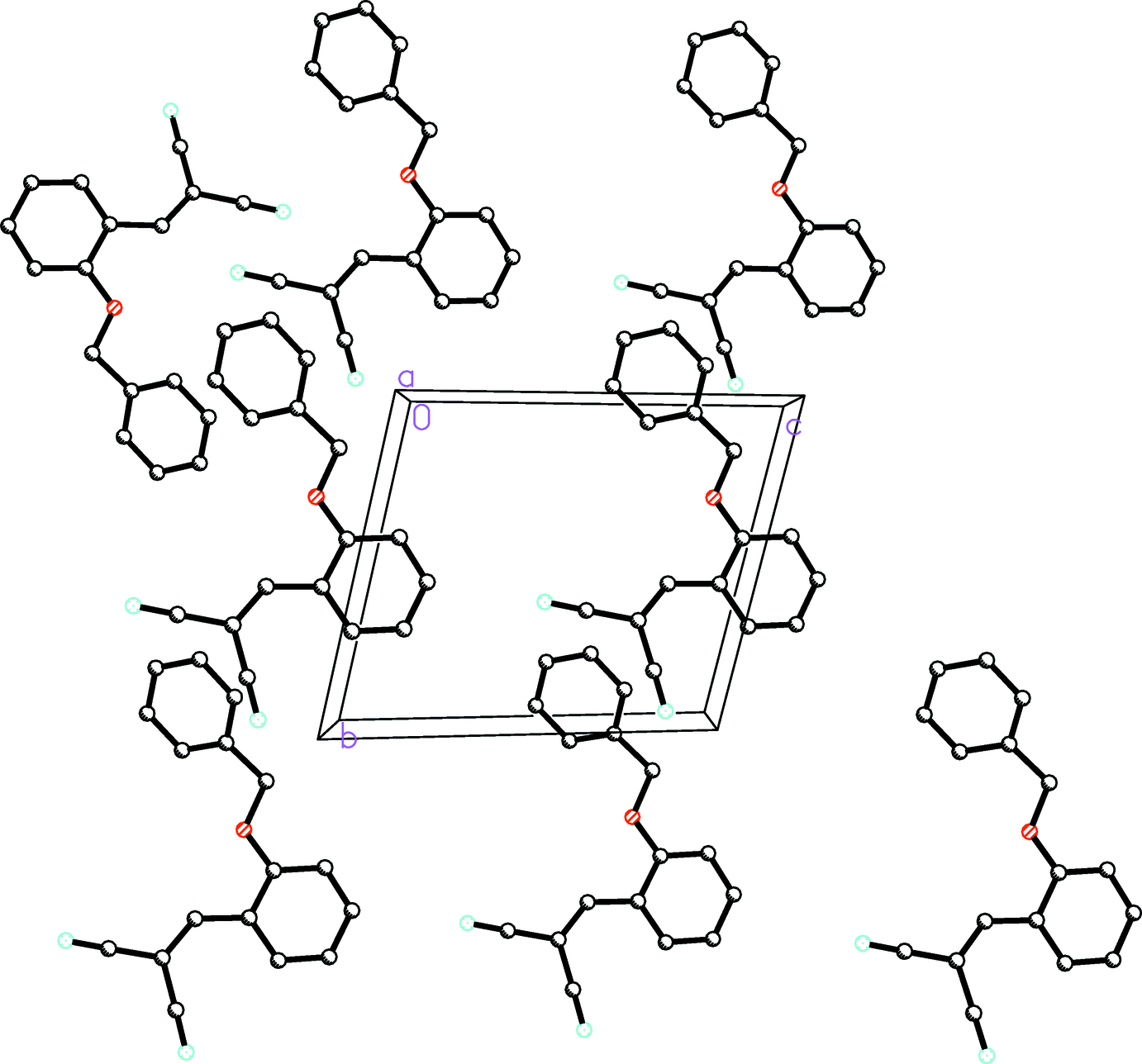

Supplement: Supplementary file 5 [file e-71-0o560-fig2.tif]
